# Remimazolam Reduces Vasopressor Use Post-Induction and During Maintenance of General Anesthesia in Patients Undergoing Laparoscopic Gynecology: A Propensity Score-Matched Analysis

**DOI:** 10.3390/jcm13216407

**Published:** 2024-10-25

**Authors:** Hyunyoung Seong, Jang Eun Cho, Seung Zhoo Yoon, Sung Uk Choi

**Affiliations:** Department of Anesthesiology and Pain Medicine, Anam Hospital, Korea University College of Medicine, Seoul 02841, Republic of Korea; hello406@kumc.or.kr (H.S.); chojangeun@korea.ac.kr (J.E.C.); yoonsz70@gmail.com (S.Z.Y.)

**Keywords:** remimazolam, vasopressor, gynecology

## Abstract

**Objectives**: Laparoscopic gynecological surgeries are commonly performed under general anesthesia and can induce cardiovascular depression and hypotension, requiring vasopressor support. Remimazolam, a novel ultra-short-acting benzodiazepine, is used to treat minimal cardiovascular depression. This study compared the hemodynamic effects of remimazolam and sevoflurane anesthesia in patients undergoing laparoscopic gynecological surgery. **Methods**: A retrospective analysis was conducted on 474 patients who underwent laparoscopic gynecological surgery at Korea University Anam Hospital between September 2021 and December 2022. The patients were categorized into two groups based on the anesthetic agent used: remimazolam or sevoflurane. Hemodynamic parameters, vasopressor use, and intraoperative variables were compared between anesthetic agents. Propensity score matching was applied to account for potential confounders, and logistic regression was utilized to assess the relationship between anesthesia type and outcomes. **Results**: Remimazolam anesthesia was linked to a significantly lower incidence of vasopressor use compared to sevoflurane-based anesthesia (3.7% vs. 19.5%, *p* < 0.0001). The odds of requiring vasopressor support were significantly lower during the post-induction and maintenance phases in the remimazolam group. Furthermore, hemodynamic stability, particularly systolic and mean arterial pressures, was better maintained with remimazolam than sevoflurane. **Conclusions**: Remimazolam provides superior hemodynamic stability and reduces the need for vasopressor support during laparoscopic gynecological surgery compared with sevoflurane.

## 1. Introduction

Laparoscopic gynecological surgeries, such as hysterectomy, myomectomy, and ovarian cystectomy, are the predominant surgeries performed in women [1]. They are usually performed under general anesthesia with a balanced anesthesia technique, commonly using sevoflurane and a remifentanil infusion for maintenance [2]. These anesthetics have yielded better results in ambulatory settings [3], with a higher percentage of patients classified as fast-track [2] than total intravenous anesthesia using propofol.

However, these anesthetic techniques can induce potentially fatal cardiovascular depression and hypotension after induction and during surgery [4,5]. Up to 77.4% of female patients undergoing gynecological surgery experience at least one hypotensive event during the surgery [6]. Therefore, anesthetic agents that do not induce cardiovascular depression or hemodynamic instability will be useful for the induction and maintenance of anesthesia.

Remimazolam, a benzodiazepine analog known for its ultrashort-acting properties and favorable pharmacokinetic profile, has been recently approved for general anesthesia in induction and maintenance [7,8]. Most importantly, remimazolam has minimal cardiovascular depressant effects and can reduce the incidence of hypotension [8,9]. Although earlier studies demonstrated superior hemodynamic stability with remimazolam than with propofol [8,9], there is limited research on its effects compared to those of sevoflurane anesthesia during gynecologic surgery. This study addressed this gap by comparing the intraoperative hemodynamic effects of remimazolam and sevoflurane anesthesia in patients undergoing gynecological surgery.

## 2. Materials and Methods

### 2.1. Patients

We conducted a retrospective analysis using data from a prospectively maintained database, reviewing 512 consecutive patients who underwent laparoscopic gynecological surgery with either sevoflurane or remimazolam anesthesia. These surgeries were performed by four different surgeons at Korea University Anam Hospital between 1 September 2021 and 31 December 2022. Among the 512 patients, patients with diseases that can affect hemodynamic stability, such as severe cardiac conditions (*n* = 8), respiratory diseases (*n* = 4), or thyroid disorders (*n* = 2), were also excluded. Exclusions were also made for cases that deviate from routine surgeries enough to affect our results, such as those who had additional procedures along with laparoscopic gynecological surgery (*n* = 10), conversions to open surgery (*n* = 1), surgeries lasting more than 4 h (*n* = 8), and post-surgery ICU admissions (*n* = 2). Incomplete data (*n* = 3) were also excluded. This left a total of 474 patients, and they were divided based on the anesthetic agent used. This retrospective review was carried out at Korea University Anam Hospital and was approved by the Institutional Review Board of Korea Univeristy Ethics Committee (protocol number: 2023AN0094). Due to the nature of the study, the requirement for informed consent was waived.

### 2.2. Anesthetic Management

The attending anesthesiologists selected the anesthetic agent according to their discretion and provided consistent anesthesia according to our institution’s protocol, which is as follows. Devices to monitor pulse oximetry, noninvasive blood pressure, and electrocardiogram were used in the operating room. Additionally, a bispectral index sensor (BIS™, Medtronic, Dublin, Ireland) was used to monitor sedation depth. Premedication included the administration of 0.1 mg of glycopyrrolate just before anesthesia initiation. In the sevoflurane-based group, anesthesia induction started with a propofol (Fresofol^®^ MCT 1%; Fresenius Kabi Austria GmbH, Graz, Austria) bolus (1.0–2.0 mg·kg^−1^) and remifentanil (Ultian, Hanlim Pharm. Co., Ltd., Seoul, Republic of Korea) infusion 0.05 µg·kg^−1^·min^−1^. To reduce sudden stimulation, a bolus of remifentanil (0.5–1 μg·kg^−1^) was used right before intubation. After confirming loss of consciousness in the patient, a 0.6–1.0 mg·kg^−1^ bolus of rocuronium (Esmeron ^®^, MSD Korea, Seoul, Republic of Korea) was administered, and an electromyography (TetraGraph™, Senzime BV, Uppsala, Sweden) was used to maintain a neuromuscular blockade of 0–2 train-of-four (TOF). After intubation, sevoflurane (Sojourn, Piramal Critical Care Inc., Bethlehem, PA, USA) was used to maintain anesthesia at an age-adjusted minimal end-tidal alveolar concentration (MAC) of 0.8–1.0, and target BIS values of 40–60. After induction, remifentanil infusion was maintained at dosages of 0.05–0.25 µg·kg^−1^·min^−1^ to control MAP within 20% of the baseline value. In the cases of persistent MBP < 65 mmHg or sudden reduction of MAP over 20% despite minimal concentrations of remifentanil, boluses of phenylephrine (50–100 μg increments) or ephedrine (4 mg increments) were administered. When continuous boluses were required, continuous phenyl or norepinephrine was used. In the cases of persistent hypertension, despite maximum concentrations of remifentanil, nicardipine (500 mg increments), an anti-hypertensive agent, was used to control blood pressure.

Remimazolam (Byfavo™ injection; Hana Pharm. Co., Ltd., Seoul, Republic of Korea) was maintained at 6 mg·kg^−1^·h^−1^ until loss of consciousness of the patient and was decreased to 1–2 mg·kg^−1^·h^−1^ after intubation in the remimazolam group. Remifentanil and neuromuscular blockage were maintained the same way as the sevoflurane group.

To control postoperative pain, a sufentanil bolus (5–10 mcg) was administered before emergence. Concurrently, flumazenil (Flunil^®^ Bukwang Pharm Co., Ltd., Seoul, Republic of Korea) was administered at 0.2–0.3 mg to reverse the effects of remimazolam. In cases where the patient did not wake up completely or regain enough consciousness to follow the instructions of the attending anesthesiologist, 0.1 mg of additional flumazenil was administered, and the total dose did not exceed 0.5 mg. The patient was extubated when capable of voluntary eye-opening and able to execute instructions of the attending anesthesiologist. The patient was transferred to the recovery room once vital signs and spontaneous respiration were stable.

### 2.3. Data Collection and Outcomes

Data on patient demographics, including age, height, weight, body mass index (BMI), American Society of Anesthesiologists (ASA) classification, and underlying diseases, were retrospectively collected from medical records. Surgical and anesthetic records were obtained on the scope of surgery, as well as hemodynamic parameters such as blood pressure and heart rate during the procedure. Other data collected included anesthesia duration, total remifentanil administered, the rescue analgesia (sufentanil) administered, average sevoflurane level administered, the flumazenil dose, and the time required for endotracheal extubation (measured from the end of surgery to the completion of extubation). Additionally, intraoperative data on fluid intake and output, blood loss, and the use of phenylephrine, ephedrine, and norepinephrine were recorded. Recovery room data, including maximum pain levels assessed using the Numerical Rating Scale, the need for additional narcotic analgesics (fentanyl), use of rescue medications for postoperative vomiting, Aldrete scores at both admission and discharge from the recovery room, and length of hospital stay after surgery, were also gathered. The primary outcome was the variation in the frequency of vasopressor use during surgery. Secondary outcomes included the frequency and odds ratio (OR) of vasopressor use across different surgical phases and hemodynamic parameters, such as systolic blood pressure (SBP), diastolic mean arterial pressure (MAP), blood pressure (DBP), and heart rate (HR), throughout the procedure.

### 2.4. Statistical Analysis

We presented continuous variables as medians with interquartile ranges (first and third quartiles) and categorical variables as numbers and percentages. Data comparisons between the two groups were made using the Mann–Whitney U test for continuous variables and the Chi-squared or Fisher’s exact tests for categorical variables.

We used logistic regression before propensity score matching to control several confounding variables and elicited propensity scores between each subject within the remimazolam and sevoflurane-based groups. Independent variables in the analysis included potential confounders such as age, height, weight, ASA classification, comorbidities (including hypertension, respiratory disease, a history of cardiovascular events, and diabetes), duration of anesthesia, intraoperative fluid balance, and type of surgery. The nearest-neighbor method was employed for PSM, with a caliper of 0.25 and a 1:1 ratio of treated to control units. A standardized mean difference (SMD) of <0.1 was considered evidence of balance between variables in the matched dataset. Subsequently, a conditional logistic regression model with stepwise variable selection was used to estimate odds ratios (ORs) across various outcomes in the matched cohort. By using binary and multivariable logistic regression, the association between independent and outcome variables was explored. A *p*-value of < 0.05 was considered indicative of significant associations with the outcome variables. Statistical analyses were conducted using SPSS version 21 (SPSS Inc., Chicago, IL, USA).

## 3. Results

### 3.1. Subsection Patient Characteristics

Before applying propensity score matching (PSM), there were 174 patients in the sevoflurane-based group (Group S) and 300 in the remimazolam group. The sevoflurane-based group had a higher occurrence of cystectomy and myomectomy procedures, along with lower intraoperative fluid balance, compared to the group that used remimazolam. Other variables were similar, as shown in Table 1.

### 3.2. Vasopressor Use

Table 2 shows the number and frequency of patients who used vasopressors in the remimazolam and sevoflurane-based group before and after PSM. Remimazolam anesthesia was significantly associated with a lower frequency of total vasopressor use (19.5% vs. 3.7%, *p* < 0.0001), which was also observed when separately analyzing the use of ephedrine (14.0% vs. 0.6%, *p* < 0.0001) and phenylephrine (8.5% vs. 3.0%, *p* = 0.039). No significant result was observed with norepinephrine due to the small number of patients.

Table 3 shows the frequency of vasopressor use according to the anesthesia phase, divided into the post-induction [induction to initiate surgery] and maintenance phases [start to end of surgery]. Remimazolam anesthesia was significantly associated with a lower frequency of total vasopressor use not only in the post-induction phase [6.7% vs. 2.4%, *p* < 0.001] but also in the maintenance phase [14.0% vs. 3.0%, *p* < 0.001]. There was no significant difference between the groups at the end of the surgical phase [end of surgery to extubation].

**Figure 1:**
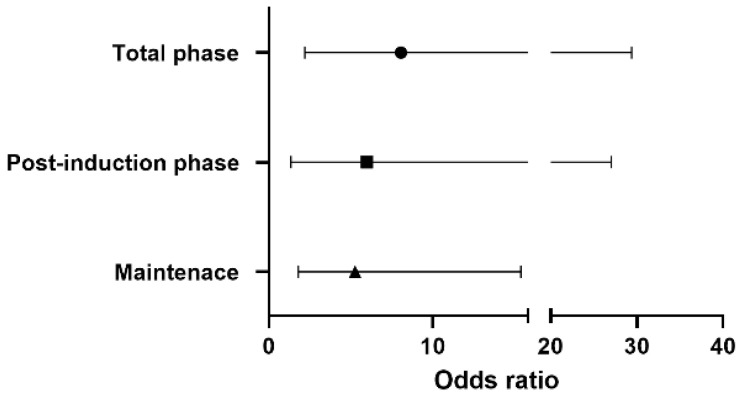
The odds ratio of vasopressor use in the sevoflurane-based group according to the phase of surgery.

Figure 1 shows the odds ratio for vasopressor use in each phase. Throughout the surgery, the risk of vasopressor use in the sevoflurane-based group was 8.06 times higher than that in the remimazolam group [OR 8.06, 95% CI 2.20–29.41, *p*-value 0.0016]. A 5.99 times higher risk of vasopressor use [OR 5.99, 95% CI 1.34–27.03, *p*-value 0.019] was observed in the post-induction phase, with 5.26 times higher [OR 5.26, 95% CI 1.80–15.38, *p*-value 0.0024] risk during maintenance in the sevoflurane-based anesthesia.

### 3.3. Factors Associated with Vasopressor Use

According to univariable and multivariable logistic regression analyses for vasopressor use, the independent variables associated with the post-induction phase were propofol as an induction agent (OR = 6.94), use of angiotensin-converting-enzyme inhibitors (ACE inhibitors) or angiotensin II receptor antagonists (ARBs) (OR = 1.002), baseline SBP (OR = 0.701), and the I/O fluid balance (OR = 0.996). During the maintenance phase, the independent variables associated with vasopressor use were sevoflurane use as maintenance (OR = 5.95), age (OR = 1.055), baseline SBP (OR = 0.663), RBC transfusion (OR = 2.049), and the I/O fluid balance (OR = 0.997). These results are summarized in Table 4.

### 3.4. Intraoperative Hemodynamics

**Figure 2:**
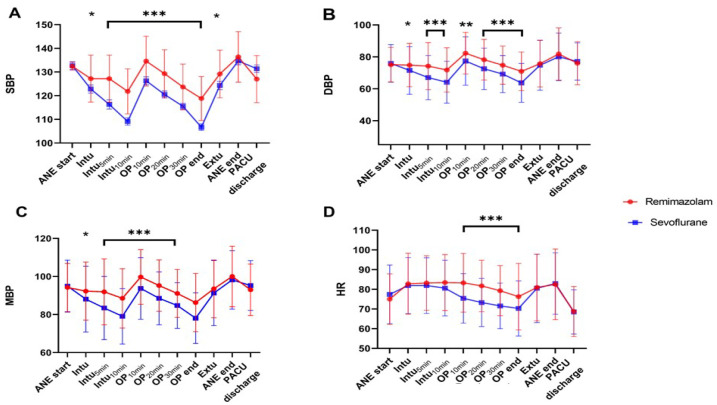
Hemodynamic parameters ((**A**); SBP, (**B**); DBP, (**C**); MBP, (**D**); HR) at different time points as the surgery progresses. In the sevoflurane group, SBP, DBP, and MAP from T2 to T6 are significantly lower than that of the remimazolam group and lower than the baseline BP. Timepoint: ANE start: Start of anesthesia, Intu: Time of endotracheal intubation, Intu_5min_: 5 min after endotracheal intubation, Intu_10min_: 10 min after endotracheal intubation, OP_10min_: 10 min after the start of surgery, OP_20min_: 20 min after the start of surgery, OP_30min_: 30 min after the start of surgery, OP end: End of surgery, Extu: Time of endotracheal extubation, ANE end: End of anesthesia, PACU discharge: Discharge from the recovery room. *, *p* < 0.05; **, *p* < 0.01; ***, *p* < 0.001.

Figure 2 shows the main hemodynamic indicators (SBP, DBP, MBP, and HR) during surgery. The remimazolam group generally maintained a better baseline BP than that of the sevoflurane-based group. As shown in Figure 2A,C, after endotracheal intubation, induction with propofol and maintenance with sevoflurane caused a rapid decrease of >20% from baseline in SBP and MAP at 5 min and 10 min after intubation, during the post-induction phase. In contrast, the decrease was not as pronounced with remimazolam. Furthermore, sevoflurane significantly decreased HR as the surgery progressed (Figure 2D), whereas remimazolam maintained it above the baseline level.

### 3.5. Perioperative Variables

Table 5 shows the recovery profiles of the two groups. There were no differences in surgery duration, anesthesia duration, and extubation time. Notably, the remimazolam group required higher doses of intraoperative remifentanil and sufentanil than those of the sevoflurane-based group [remifentanil, 0.5 vs. 0.9 mcg; sufentanil 6.5 mcg vs. 10.8 mcg, all *p* < 0.0001]. The NSR score in the recovery room was higher in the remimazolam group [3.5 vs. 3.8, *p* = 0.002], and the requirement for rescue analgesics was also higher in the remimazolam group [fentanyl, 18.7 vs. 37.8 mcg, *p* < 0.0001]. Additionally, fewer patients in the remimazolam group experienced post-operative nausea/vomiting, and the proportion of patients requiring post-operative nausea and vomiting rescue drugs was also lower in the remimazolam group.

## 4. Discussion

In summary, remimazolam anesthesia in laparoscopic gynecological surgery decreased the frequency of vasopressor use [19.5% vs. 3.7%, *p* < 0.0001] and maintained hemodynamic stability more effectively than sevoflurane-based anesthesia. Sevoflurane anesthesia with propofol as hypnotics increased the risk of total vasopressor use by 8.06 times [OR 8.06, 95% CI 2.20–29.411, *p* = 0.0016] compared with remimazolam in the post-induction and maintenance phases. Factors that reduced the risk of intraoperative vasopressor use were the use of remimazolam, high baseline SBP, and an increased I/O fluid balance. In contrast, factors that increased the risk of vasopressor use included ACEi/ARB use, age, and transfusion.

Since being approved for use as a general anesthetic in Japan and Korea in 2020, remimazolam has been commonly used in practice and for research [10,11]. In addition to the advantages of having a rapid onset and an organ-independent metabolism, this drug has shown hemodynamic stability compared to other anesthetics [12]. Remimazolam has shown safety, efficacy, and ease of use in inducing and maintaining anesthesia, particularly in patients undergoing cardiac surgery and high-risk cardiovascular patients undergoing non-cardiac surgery [12]. Not much is known about the mechanism of its hemodynamic stability. However, previous studies have shown that remimazolam maintains hemodynamic stability by maintaining cardiac output (CO) [13,14] or by stabilizing systemic vascular resistance (SVR) [15] superior to propofol. In a study that compares remimazolam to sevoflurane, remimazolam was shown to increase cardiac index 30 min after incidences of pneumoperitoneum during robotic gastrectomy [16]. In our research, there are limitations in discussing the mechanism by which remimazolam maintains hemodynamic stability as CO, SVR was not studied, but given that the blood pressure fluctuations were similar to those observed in the previous study [16], it is thought that such hemodynamic stability comes from the mechanism that remimazolam maintains better cardiac output than sevoflurane. Interestingly, due to the low expression of the γ subunit, cardiac-specific GABA_A_ receptors show weak binding to benzodiazepines [17], which may be the reason benzodiazepines have little effect on cardiac contractility. However, because this study is a small sample-sized ex vivo study, additional research is needed to confirm the effect of remimazolam on CO.

Intraoperative hypotension is associated with various morbidities. In high-risk patients, it has been reported that if systolic blood pressure (SBP) falls more than 41–50 mmHg below baseline for more than 5 min, the likelihood of developing myocardial infarction increases more than threefold (OR = 3.42) [18]. In a study regarding non-cardiac surgery patients, the odds ratios of major cardiac or cerebrovascular events within 30 days after surgery were 12%, 17%, and 26% when mean arterial pressure (MAP) was ≤75, ≤65, and ≤55 mmHg, respectively [19]. The important point is that such hypotension is a modifiable factor that can be prevented, and it is necessary to maintain stable blood pressure through the use of vasopressors in cases of intraoperative hypotension.

As noted by Südfeld et al. [20], general anesthesia-related hypotension has different underlying causes depending on the surgical phase: post-induction hypotension (PIH) and early intraoperative hypotension (eIOH). PIH, primarily occurring within 20 min after induction and before skin incision, affects 10.3–66.96% of patients [21]. This is largely attributable to the effects of vasodilation and cardiac suppression induced by general anesthetics. Several reported predictors of PIH include higher fentanyl or propofol dose used as an induction agent, female sex, lower pre-induction BP, ASA-PS III–V, and an age of over 50 years [22,23]. As commonly used anesthetics, propofol, and thiopental have 5.3- and 4.87 times higher risk of inducing PIH, respectively, than ketamine [21]. Previous studies have also shown that propofol administration can reduce MAP by 26% even in ASA I–II patients [24], highlighting the need for alternative drugs in high-risk patients. In our study, remimazolam anesthesia decreased the risk of PIH by 0.223 times compared with sevoflurane-based anesthesia using propofol as a hypnotic. Therefore, in patients expected to have difficulty in controlling blood pressure during surgery or high-risk patients, if individualized anesthesia is provided through the use of remimazolam, it is expected to yield favorable outcomes for the patient’s overall result.

IOH can be influenced by surgery-related factors, such as major hemorrhage, hemodynamically relevant surgical maneuvers, or secondary systemic inflammatory responses [20]. Through multivariable analysis, our study found that using remimazolam as a maintenance anesthetic reduced the risk of vasopressor use by 0.232 times compared with sevoflurane. Our results also indicate that age, a low baseline SBP, negative I/O fluid balance, and RBC transfusion were risk factors associated with IOH, consistent with previous studies [20,22,25].

Additionally, the remimazolam group required higher amounts of opioids during and after surgery and had higher postoperative pain scores. This finding suggests a potential link between remimazolam and post-operative hyperalgesia, warranting further investigation. Remifentanil is a commonly used ultra-short-acting opioid analgesic that can control responses to stimuli during surgery and enable rapid recovery after general anesthesia. However, when such short-acting opioids are administered at high concentrations (over 0.2 µg·kg^−1^·min^−1^), they may be associated with acute opioid tolerance and/or opioid-induced hyperalgesia. This may cause secondary hyperalgesia, increase postoperative pain, and increase postoperative opioid use [26,27]. Previous studies have suggested that high concentrations of remifentanil are needed when remimazolam is used as the general anesthetic [16,28,29], but those studies lack mention of postoperative opioid requirements or postoperative hyperalgesia. According to our analysis, the remifentanil dose required during surgery in the remimazolam group was approximately twice as high (0.2 µg·kg^−1^·min^−1^) compared to those of the sevoflurane-based group (0.1 µg·kg^−1^·min^−1^), which may have had an impact on higher postoperative opioid requirements. However, a study by Lee et al. that shows results contradictory to those of our study shows that there was no difference in opioid-induced hyperalgesia between anesthesia using remimazolam with high dose remifentanil (0.3 µg·kg^−1^·min^−1^) and desflurane with low dose remifentanil (0.05 µg·kg^−1^·min^−1^) [30]. Taking this into consideration, additional large-scale studies are necessary to analyze the effect of remimazolam and remifentanil on postoperative pain, and it would be necessary to confirm the actual pain scores of the patients using a nociceptive index during and after surgery.

This study has several limitations. First, it is retrospective in nature. However, the use of propensity score matching ensured that the study groups were well-balanced, lending credibility to our findings regarding the hemodynamic benefits of using remimazolam as the primary hypnotic agent during general anesthesia. Second, the study was conducted at a single institution, and factors such as variations in case volume and surgical expertise may have influenced aspects like surgery duration, which could affect the generalizability of our results. Thirdly, how hemodynamic stability due to the use of remimazolam affects postoperative outcomes was not analyzed in this study. Low intraoperative blood pressure can cause several complications, including acute kidney injury (AKI), myocardial injury, and mortality [31,32], but studies to date have not been able to prove that the use of remimazolm can improve AKI or mortality [33,34]. However, because the sample size of the previous papers is too small to confirm postoperative complications, further larger-scale studies are needed to confirm whether hemodynamic stability due to remimazolam has a favorable effect on postoperative outcomes. Lastly, the majority of patients in our study had an ASA physical status of II or III. The findings may differ in other populations, especially since our study did not include patients with severe liver disease or end-stage renal failure, conditions known to influence the pharmacokinetics of remimazolam.

## 5. Conclusions

In conclusion, our results indicate that remimazolam anesthesia provides superior hemodynamic stability compared to sevoflurane-based anesthesia when used as a general anesthetic. Therefore, remimazolam may be particularly advantageous for patients who are at risk of hypotension during laparoscopic gynecological procedures.

## Figures and Tables

**Table 1:** Patient demographics and baseline characteristics between two groups.

	Before PSM			After PSM		
Variable	Group S (*n* = 174)	Group R (*n* = 300)	*p*-Value	Group S (*n* = 164)	Group R (*n* = 164)	*p*-Value
Age (years)	44.0 (34.0, 52.0)	46.0 (36.5, 52.0)	0.4679 ^1^	44.0 (34.0, 52.0)	43.0 (33.5, 50.0)	0.3869 ^2^
Height (cm)	160.0 (156.0, 164.0)	159.0 (155.0, 164.0)	0.2682 ^1^	160.0 (156.0, 164.0)	160.0 (156.0, 164.0)	0.7757 ^2^
Weight (cm)	59.0 (53.0, 67.0)	59.0 (53.0, 67.0)	0.9215 ^1^	59.0 (53.0, 67.0)	58.0 (53.0, 67.5)	0.8164 ^2^
BMI (kg/m^2^)	23.2 (20.8, 26.7)	23.6 (21.0, 26.8)	0.5492 ^1^	23.1 (20.8, 26.8)	23.6 (20.9, 26.8)	0.7935 ^2^
ASA classification, *n* (%)			0.3271 ^3^			N.A ^2^
1	6 (3.5%)	5 (1.7%)		3 (1.8%)	3 (1.8%)	
2	149 (86.1%)	270 (90.0%)		149 (90.9%)	149 (90.9%)	
3	18 (10.4%)	25 (8.3%)		12 (7.3%)	12 (7.3%)	
Comorbidities, *n* (%)						
Hypertension	22 (12.6%)	52 (17.3%)	0.1751 ^3^	21 (12.8%)	24 (14.6%)	0.5316 ^5^
Asthma, COPD	3 (1.7%)	6 (2.0%)	1.0000 ^4^	3 (1.8%)	2 (1.2%)	0.6547 ^5^
CVA	3 (1.7%)	1 (0.3%)	0.1424 ^4^	2 (1.2%)	0 (0.0%)	0.1573 ^5^
Diabetes	11 (6.3%)	20 (6.7%)	0.8836 ^3^	10 (6.1%)	12 (7.3%)	0.6698 ^5^
Surgical procedure, *n* (%)			0.0155 ^4^			0.1028 ^6^
Ovarian cystectomy/tumor resection	96 (55.2%)	141 (47.0%)		89 (54.3%)	86 (52.4%)	
Myomectomy	27 (15.5%)	31 (10.3%)		25 (15.2%)	20 (12.2%)	
TLH/Laparoscopic-assisted vaginal hysterectomy	48 (27.6%)	124 (41.3%)		48 (29.3%)	56 (34.1%)	
Laparoscopy for others	3 (1.7%)	4 (1.3%)		2 (1.2%)	2 (1.2%)	
ANE duration (min)	132.8 (54.49)	128.3 (45.58)	0.8086 ^1^	132.2 (54.57)	123.5 (45.39)	0.1277 ^2^
Input—Output	190.0 (80.0, 320.0)	275 (200.0, 300.0)	0.0042 ^1^	210 (150.0, 190.0)	225.0 (80.0, 350.0)	0.1153 ^2^

Values are presented as the median (first quartile, third quartile) or number of patients (%). ^1^ Wilcoxon rank sum *p*-value; ^2^ Wilcoxon’s signed rank *p*-value; ^3^ Chi-Square *p*-value; ^4^ Fisher’s exact *p*-value; ^5^ McNemar Chi-Square *p*-value; ^6^ Symmetry *p*-value. PSM, Propensity Score Matching; S, sevoflurane-based; R, remimazolam; BMI, body mass index; ASA, American Society of Anesthesiologists; COPD, chronic obstructive pulmonary disease; CVA, cardiovascular accidents; TLH, total laparoscopic hysterectomy; ANE, anesthesia.

**Table 2:** Frequency of vasopressor use according to type of vasopressors, before and after PSM.

	Before PSM			After PSM		
Variables, *n* (%)	Group S (*n* = 174)	Group R (*n* = 300)	*p*-Value	Group S (*n* = 164)	Group R (*n* = 164)	*p*-Value
Intraop total vasopressor	34 (19.5%)	20 (6.7%)	<0.0001 ^1^	32 (19.5%)	6 (3.7%)	<0.0001 ^3^
Intraop ephedrine	24 (13.8%)	6 (2.0%)	<0.0001 ^1^	23 (14.0%)	1 (0.6%)	<0.0001 ^3^
Intraop phenylephrine	16 (9.2%)	16 (5.3%)	0.1062 ^1^	14 (8.5%)	5 (3.0%)	0.0389 ^3^
Intraop NE	1 (0.6%)	0 (0.0%)	0.3671 ^2^	1 (0.7%)	0 (0.0%)	0.3173 ^3^

Intraop, intraoperative; NE, norepinephrine. ^1^ Chi-Square *p*-value; ^2^ Fisher’s exact *p*-value; ^3^ McNemar Chi-Square *p*-value.

**Table 3:** Frequency of vasopressor according to the phase of surgery, before and after PSM.

	Before PSM			After PSM		
Phase, *n* (%)	Group S (*n* = 174)	Group R (*n* = 300)	*p*-Value	Group S (*n* = 164)	Group R (*n* = 164)	*p*-Value
Post-induction	12 (7.1%)	5 (1.7%)	0.0026 ^1^	11 (6.7%)	3 (2.4%)	<0.0001 ^3^
Maintenance	24 (14.1%)	11 (3.7%)	<0.0001 ^1^	23 (14.0%)	5 (3.0%)	<0.0001 ^3^
End of surgery	1 (0.6%)	3 (1.0%)	1.000 ^2^	1 (0.6%)	1 (0.6%)	1.000 ^3^

^1^ Chi-Square *p*-value; ^2^ Fisher’s exact *p*-value; ^3^ McNemar Chi-Square *p*-value.

**Table 4:** Independent variables and variable categories were significantly associated with hypotension during the post-induction and maintenance phases.

Variable	Level	Univariable Logistic Regression				Multivariable Logistic Regression (Stepwise)			
				95% CI for OR				95% CI for OR	
		*p*-Value	OR	Lower Limit	Upper Limit	*p*-Value	OR	Lower Limit	Upper Limit
**Post-Induction**									
Induction	Propofol	0.0056	4.48	12.98	1.55	0.0007	6.94	2.26	21.74
	Remimazolam	-	1	-	-				
Age		0.4145	1.015	0.980	1.051				
Height		0.4841	0.906	0.688	1.194				
Weight		0.8841	1.091	0.338	3.52				
BMI		0.7926	1.015	0.911	1.130				
Hypertension		0.6562	0.712	0.159	3.18				
Use of ACEi/ARB		0.0873	1.001	1.000	1.003	0.0227	1.002	1.000	1.003
Induction duration		0.2832	1.035	0.972	1.101				
Baseline SBP		0.0642	0.740	0.538	1.018	0.0392	0.701	0.500	0.983
Change of Hb		0.4841	0.906	0.688	1.194				
ASA classification *	2	0.9036	0.831	0.041	16.740				
	3	0.6472	2.090	0.089	49.165				
	1	-	1	-	-				
Input-Output fluid balance		0.1435	0.997	0.994	1.001	0.0229	0.996	0.992	0.999
**Maintenance**									
Maintenance	Sevoflurane	0.0001	4.310	9.091	2.058	<0.0001	5.95	13.51	2.632
	Remimazolam	-	1	-	-				
Age		0.012	1.033	1.007	1.059	0.0006	1.055	1.023	1.088
BMI		0.2556	1.043	0.970	1.123				
ASAclass *	2	0.6939	1.819	0.092	35.804				
	3	0.4311	3.464	0.157	76.348				
	1	-	1	-	-				
Induction duration		0.1844	1.031	0.985	1.079				
OP Time		0.0111	1.008	1.002	1.014				
Baseline SBP		0.0002	0.690	0.570	0.836	0.0002	0.663	0.532	0.826
Change of Hb		0.0502	0.792	0.628	1				
RBC transfusion		0.0020	2.406	1.380	4.194	0.0275	2.049	1.083	3.875
Hypertension		0.093	1.990	0.892	4.444				
Use of ARB		0.0458	1.001	1	1.002				
Input-Output fluid balance		0.1318	0.998	0.996	1.001	0.0118	0.997	0.994	0.999

* Penalized logistic regression in univariate analysis. CI, confidence interval; OR, odds ratio; ACEi, angiotensin-converting-enzyme inhibitors; ARB, angiotensin II receptor antagonists. For Post-induction, ROC: 0.832 (raw data), 0.7765 (cross-validation). For the maintenance phase, ROC: 0.825 (raw data), 0.7892 (cross-validation).

**Table 5:** Intraoperative and Recovery room variables.

	Before PSM			After PSM		
Variables	Group S	Group R	*p*-Value	Group S	Group R	*p*-Value
**Intraoperative variables**						
OP time (min)	95.5 (52.20)	90.6 (44.83)	0.6411 ^1^	94.9 (52.44)	86.8 (44.45)	0.1195 ^2^
Extubation time (min)	6.1 (3.21)	6.0 (3.34)	0.8971 ^1^	6.1 (3.20)	5.8 (3.52)	0.1210 ^2^
Intraop Remimazolam total dose (mg)	0.0 (0.00)	143.7 (64.94)	<0.0001 ^1^	0.0 (0.00)	138.0 (67.74)	<0.0001 ^2^
Intraop Remifentanil total dose (μg)	0.5 (0.32)	0.9 (0.50)	<0.0001 ^1^	0.5 (0.30)	0.9 (0.52)	<0.0001 ^2^
Intraop Remifentanil average dose (μg/kg/min)	0.1 (0.06)	0.2 (0.08)	<0.0001 ^1^	0.1 (0.05)	0.2 (0.09)	<0.0001 ^2^
Intraop Sufentanil (μg)	6.5 (4.64)	11.0 (5.78)	<0.0001 ^1^	6.5 (4.74)	10.8 (6.25)	<0.0001 ^2^
**Recovery room variables**						
Maximal pain score (NRS)	3.5 (0.88)	3.8 (0.88)	<0.0001 ^1^	3.5 (0.91)	3.8 (0.88)	0.0020 ^2^
Rescue analgesics (Fentanyl, μg)	18.7 (32.72)	37.6 (37.32)	<0.0001 ^1^	18.7 (32.34)	37.8 (37.20)	<0.0001 ^2^
Postop nausea/vomiting (PONV)	0.2 (0.36)	0.0 (0.17)	<0.0001 ^1^	0.2 (0.37)	0.0 (0.17)	0.0001 ^2^
PONV rescue drug	0.2 (0.39)	0.0 (0.20)	<0.0001 ^1^	0.2 (0.39)	0.0 (0.22)	0.0003 ^2^
Aldrete score						
At arrival	9.0 (0.15)	9.0 (0.06)	0.1436 ^1^	9.0 (0.16)	9.0 (0.00)	0.3173 ^2^
Before discharge	10.0 (0.00)	9.9 (0.82)	0.1871 ^1^	10.0 (0.00)	9.9 (0.78)	0.1797 ^2^
Discharge (POD, day)	2.6 (1.04)	2.5 (1.03)	0.1040 ^1^	2.6 (1.03)	2.4 (1.01)	0.2179 ^2^

Values are presented as the mean (SD) or number of patients (%). ^1^ Wilcoxon rank sum *p*-value; ^2^ Wilcoxon’s signed rank *p*-value. PSM, Propensity Score Matching; OP, Operation.

## Data Availability

Due to privacy concerns, the data presented in this study are available upon request from the corresponding author.
